# The Oncology Clinical Nurse Specialist: A Rapid Review of Implementation Models and Barriers around the World

**DOI:** 10.3390/curroncol30080538

**Published:** 2023-08-05

**Authors:** Ori Kapra, Noam Asna, Mazal Amoyal, Osnat Bashkin, Keren Dopelt

**Affiliations:** 1 Department of Public Health, Ashkelon Academic College, Ashkelon 78211, Israel; orikapra@outlook.com (O.K.); osnatba@edu.aac.ac.il (O.B.); 2 Oncology Institute, Shaare Zedek Medical Center, Jerusalem 91031, Israel; noamas@szmc.org.il; 3 Palliative Care Unit, Barzilai Medical Center, Ashkelon 78306, Israel; mazalam@bmc.gov.il; 4 School of Public Health, Faculty of Health Sciences, Ben Gurion University of the Negev, Beer Sheva 84105, Israel

**Keywords:** clinical nurse specialist, advanced nurse practitioner, oncology, rapid review

## Abstract

The role of a clinical nurse specialist in oncology varies greatly between healthcare systems, and implementing this healthcare role with its multifaceted and co-existing responsibilities may prove challenging. While already integrated into healthcare systems and services in several European countries, Asia, Canada, and the United States, other countries are just beginning to develop clinical nursing specialties. The current study aims to provide healthcare policymakers with up-to-date evidence that focuses on the diverse modes of oncology clinical nurse specialist role implementation across several healthcare systems and pertinent implementation challenges as described in the literature. A rapid evidence assessment was carried out in order to provide policymakers with a rigorous review in a condensed timescale. Initially, only items in the English language were included, and “grey literature” was excluded. We searched PubMed between 1 January 2022 and 28 February 2022 and two independent scholars reviewed items. Based on 64 papers, both non-scientific and papers that met the initial criteria of the rapid review, we describe the modes of implementation of the oncology clinical nurse specialist in the United States, Canada, United Kingdom, Japan, Brazil and Australia. Barriers to implementation include conflicts around role boundaries, skepticism and lack of organizational support, as well as fears that oncology clinical nurse specialists will “encroach” on doctors’ powers. In contrast, an oncology clinical nurse specialist is found to be universally more accessible to patients and their families and can help physicians deal with difficult workloads, among other advantages. Conclusions: This role offers a myriad of gains for cancer patients, oncology physicians, and the healthcare system. The literature demonstrates that it is a necessary role, albeit one that brings specific implementation challenges.

## 1. Introduction

A clinical nurse specialist is “an Advanced Practice Nurse who provides expert clinical advice and care based on established diagnoses in specialist clinical fields of practice” [1]. The role includes diverse components, operating together, including providing information, counseling and support; managing care; and engaging in research, teaching, and service developments, usually focusing on leadership and education [2,3]. The requirements and training for clinical nurse specialist roles vary significantly between countries and even between different states in the United States. Responsibilities, supervision, and the ability to work independently without a doctor’s supervision also vary between countries.

There have been several attempts to analyze the clinical nurse specialist role, with the aim of creating a normative framework to organize its diverse, multifaceted, and co-existing responsibilities. The United Kingdom’s Royal College of Nursing [4] has proposed that, depending on clinical specialty, a clinical nurse specialist role should be 67% clinical, 21% administration, 6% education, 4% research, and 2% consultation. Bryant-Lukosius et al. [5] observed that oncology advanced practice nurses in Ontario undertake roles combining all the above components with a high degree of role variability, yet, on average, allocate most of their time to providing direct clinical care (62.7%), education (13.0%), organizational leadership (11.5%), research (6.7%), and scholarly and professional development (6.5%).

Internationally, the absolute minimum requirement for oncology clinical nurse specialist roles is a master’s degree in nursing, which includes courses in oncology. Healthcare systems in different countries vary in their additional requirements for oncology clinical nurse specialist roles. The Japanese certification board stipulates that oncology clinical nurse specialist candidates must have 5 years of clinical experience (including 3 years in a defined specialty area, at least one post-graduation), and that oncology clinical nurse specialists must reapply to the certification board every 5 years for license renewal [6]. In contrast, in the United States, standardized clinical experience requirements are seldom stipulated, a situation that has prompted national-level strategies to help newly graduated oncology clinical nurse specialists who have just arrived in an oncology clinical setting, including a web-based training program [7].

While the role of the clinical nurse specialist is integrated into healthcare systems and services in several European countries, Asia, Canada, and the United States, other countries are just beginning to develop clinical nursing specialties [8]. Many studies suggest that oncology clinical nurse specialists with experience of treating cancer patients likely have the most suitable skillset to provide an efficient and dignified therapeutic response to cancer patients in their lengthy, complex, and often frustrating journeys. For example, the assistance of an oncology clinical nurse specialist to help meet the goals of rapid cancer diagnosis and treatment is viewed as essential to the United Kingdom’s National Health Service [9].

Several innovative national strategies have placed oncology clinical nurse specialists as leaders of national clinic branches for treating cancer patients. This move stems from a desire to give them as much autonomy as possible and to make the oncology clinical nurse specialist service accessible. It also recognizes the efficacy—as backed by research—of managed care by oncology clinical nurse specialists [10], with specialist physician assistance provided remotely or by doctors on rotation [11]. Such specialist nurses are given a great deal of autonomy in clinical decision-making, are often experts in managing cancer symptoms [12], and have the authority to prescribe drugs. These roles dynamically combine working independently with teamwork in hospital, community, and academic settings, with the role described as “cross-sectional” [13]. Furthermore, there have been recent calls to expand the recognition of the oncology clinical nurse specialist as a gold standard in multidisciplinary teams for treating cancer patients due to, among other reasons, oncology clinical nurse specialists having the most detailed knowledge of complex patient situations that involve different professionals [14].

Alongside the advanced implementation model of the oncology clinical nurse specialist, which challenges the traditional view that oncology nurses are essentially assistants to the oncologist, a comparative global study of the implementation of this role reveals a specialty situated within complex processes across global geopolitical spaces. An analysis by the World Health Organization found that in Europe and North America, there are about 31 different certifications and degrees awarded to nurses in each region, whereas in Southeast Asia or the Middle East, there are only about 10 different certifications awarded in total [15]. In low- and middle-income countries, where cancer mortality rates are greater compared to high-income countries, it has been suggested that expanding the powers of oncology nurses may be highly beneficial in addressing the increase in the global cancer burden [13]. However, although nurses who treat cancer patients make up the majority of oncology staff in those countries, there are still gaps in the required skills [16], and oncology nursing education, practice environment, and role opportunities are described as de facto, not meeting the growing need for the role [17]. These skills gaps within the workforce, in particular among oncology nursing staff in low- and middle-income countries, continue alongside the focused efforts of the World Health Organization, professional organizations, and other bodies to improve oncology nursing (e.g., through the establishment of International Nurses Day and International Year of the Nurse), and warnings that government health insurance is powerless to improve cancer survival rates in the absence of competent oncology nursing teams [15,17].

Many issues, such as conflicts around role boundaries, implementation, lack of resources/systemic support, fears that oncology clinical nurse specialists will “encroach” on doctors’ powers, and the large variations between different healthcare systems in defining the oncology clinical nurse specialist role and the training required for it, limit the role’s potential and reduce its important contribution to quality oncology care [18]. For example, one study conducted in Canada demonstrated that stakeholders, decision makers, regulators and members of the healthcare team have a poor understanding of the role of the clinical nurse specialist [19]. This misunderstanding directly causes job dissatisfaction among clinical nurse specialists, who may decide to subsequently leave [20]. Reviewing the development of the oncology clinical nurse specialist role in different countries is particularly important in order to plan strategies to address the increase in oncology patients and the shortage of doctors. Inequality in service provision to cancer patients in peripheral areas and professional conflicts are affected, among other things, by the gender gaps that mar the development of the oncology clinical nurse specialist role in various countries.

This literature review aims to explore the implementation experience of healthcare systems around the world with the oncology clinical nurse specialist, paying particular attention to barriers that have manifested and that pertain to the role’s unique characteristics. In the face of rapid changes to the oncology healthcare workforce, and with the aim of providing healthcare policymakers with up-to-date evidence in a timely manner, this paper seeks to provide a complete literature review over a four-month period. It focuses on the diverse modes of oncology clinical nurse specialist role implementation and the pertinent implementation challenges described in the literature.

## 2. Methods

### 2.1. Research Tool

Rapid Evidence Assessment is a variation of a systematic review that balances time constraints with considerations of bias. It offers rigorous reviews in a condensed timescale [21]

The dedicated search period was limited to two out of the four months devoted to the entire study. Electronic literature searches of PubMed between 1 January 2022 and 28 February 2022 were performed. The search terms included each of the following individually and in combination: “*clinical nurse specialist*”, “*oncology nurse*”, and “*cancer nurse*”. No other data sources were included initially. To prevent any potential bias arising from the time-based selection of publications, it was stipulated that only more experienced doctoral students or doctoral graduates carry out the search and selection.

### 2.2. Inclusion and Exclusion Criteria

The PCC method (Population/Participants-Concept-Context) to identify the main concepts in the primary review question was used to inform the search strategy and the definition of inclusion criteria [22] (Table 1). The primary review question was “How do healthcare systems integrate the role of the oncology clinical nurse specialist globally and what difficulties did their encounter?”.

The papers included were limited post hoc to studies published after December 2001. This strategy was adopted in order to facilitate a modern understanding of the subject matter seeing that the wealth of available papers was sufficient for the scope of the rapid review. We initially excluded “grey literature”, such as unpublished reports, dissertations and theses. Only papers in English were included in this stage. Publications regarding nursing in oncology that were unrelated to the role of the oncology clinical nurse specialist were not included. Studies dealing solely with projects and initiatives led by oncology clinical nurse specialists were scanned but ultimately not included, because they were categorically of small scale and presented subjective and often tangential perspectives within the context of the rapid review.

### 2.3. Data Appraisal

Two independent scholars reviewed a list of articles selected for inclusion and signified their approval or disapproval of each item based on their relevance to the rapid review. Consensually irrelevant items were marked for exclusion, and controversial items were discussed until a consensus was reached. At this stage, we positively appraised published peer-reviewed systematic reviews and large population-based studies. We gave special attention to pertinent integrative reviews that already used an explicit data appraisal system.

### 2.4. Narrative-Based Data Synthesis

We grouped healthcare-system-specific data using a country index, setting the stage for a globally focused narrative. For countries where our search yielded a relative wealth of relevant studies, mostly studies receiving high appraisal in the previous step, were included in synthesis (e.g., we excluded smaller studies and/or case studies, papers with unclear and/or less than stellar methodology, and slightly tangential topics such as research on sub-specialties of oncology clinical nurse specialist). The study selection was less stringent for countries that yielded fewer results. At this stage, we selected the countries to be reviewed—the United States, Canada, the United Kingdom, Japan, Brazil, and Australia. The synthesis was conducted on 4 systematic reviews, 14 non-systematic reviews, and 21 observational studies, 2 of which are large (national) population-based studies. Additional papers were later identified by searching reference lists from retrieved papers.

## 3. Results

The results are presented as a narrative synthesis, organized by healthcare system, based on 64 papers that met the criteria of the rapid review. In addition to various sources later retrieved by searching the reference lists of the selected papers, the final paper list includes 4 systematic reviews, 13 non-systematic reviews, and 19 observational studies (Table 2).

### A Picture of the Global Situation Regarding the Role of the Clinical Nurse Specialist in Oncology

- The United States

Oncology clinical nurse specialists in the United States (often referred to as oncology nurse practitioners) are graduates with a master’s degree in nursing with expertise in oncology and the treatment of a defined patient population [33]. They are trained to provide comprehensive care, including various expert-level skills, such as taking a patient’s medical history, ordering and interpreting medical examinations, making a diagnosis, providing acute and chronic treatment, managing cancer symptoms, and prescribing pharmacological and non-pharmacological treatments (chemotherapy, narcotic drugs, psychotherapy, and support frameworks) [45]. The scope of an oncology clinical nurse specialist’s role is not defined by the work environment but by the needs of the patient [33]. Oncology clinical nurse specialists are exceptional among healthcare providers in terms of their ability to establish a high degree of trust with patients, including forming relationships that last for many years [40]. This high level of trust, which is also expressed in terms of high satisfaction ratings by patients who are treated by oncology clinical nurse specialists [28], provides specialist nurses with special opportunities to implement behavioral modifications in the treatment and prevention of cancer while being sensitive to a patient’s psychosocial condition [46]. These include behavior modifications that are perceived by patients and healthcare services as impossible to implement or as not being important enough [31]. In addition to the reported patient satisfaction rates, it seems that in the United States, there is also an economic rationale for expanding and improving the oncology clinical nurse specialist role, since such nurses can not only administer high-quality treatment and potentially reduce high patient costs, but are also less expensive to hire compared to doctors [28]. In the vast majority of cases, advanced nursing degrees in the United States offer only ancillary courses in oncology, and this specialty is not the main focus of the degree. This is reflected in surveys, which indicate that that the majority of nursing students at the start of their careers do not feel qualified to deal with clinical issues such as chemotherapy, toxicity in drug administration, or emergency treatment [34]. The National Cancer Institute has funded the establishment of network-based programs, and this literature review aims to bridge this gap by assisting oncology clinical nurse specialists who have just entered the oncology care environment [7]. To address the issue of nonstandard training, efforts are being made to create a national standard for the certification and licensing of nurse experts, which includes the renewal of oncology clinical nurse specialist licenses. However, as of 2018, fewer than half of all states in the United States operate according to a uniform standard for registering specialist clinical nurses [45].

- Canada

According to the Canadian Association of Nurses in Oncology [47], an oncology clinical nurse specialist is “a licensed nurse with at least a master’s degree who has acquired in-depth knowledge and clinical experience in oncology”. The Canadian Nurses Association distinguishes between clinical nurse specialists (an established profession in Canada that developed within a hospital setting no later than the 1960s in a variety of specialties, including oncology) and a relatively new type of specialist clinical nurse known as an advanced nurse practitioner, a role characterized by a combination of different degrees of professional knowledge including education, organizational leadership, research, direct patient care, and professional learning and development [47]. This type of oncology clinical nurse specialist role, unlike the older clinical nurse specialist roles, is not limited to the organizational boundaries of the hospital but has a higher level of autonomy and is an expression of innovation that only began to emerge in the 21st century [5]. The two subtypes of oncology nurse in Canada (clinical nurse specialist and advanced nurse practitioner) both shed light on the oncology clinical nurse specialist profession since the Canadian case demonstrates that even countries with an apparently rich history of implementing this role have only recently responded to innovative applications of it, similar to countries that first need to make improvements to oncology nursing [5]. New studies have ascertained that the Canadian healthcare system, like those in most Western countries, faces difficulties in implementing innovations in the oncology clinical nurse specialist role in ways that also affect the satisfaction of clinical nurses specializing in oncology in their roles [20]. The main challenges that have been identified include poor understanding of the role by decision makers, lack of clarity about the role, lack of support from management, and misunderstandings of medical staff [19,48]. In studies that preceded the implementation of nursing reforms in European countries, such as in Ireland, it is often argued that the Canadian experience demonstrates that the oncology clinical nurse specialist is an established role [49], while ignoring calls to address new challenges that would allow better definition, planning, and implementation of the clinical oncology specialism in nursing [5]. A comprehensive poll conducted in the province of Ontario shows that of the 77 oncology clinical nurse specialists working in the province, 33% were considering leaving their profession or were actively looking for a new job, and there was agreement among administrators in the province that “new ways of working” were needed in order to establish a cancer care system that was high quality, efficient, and patient-centered [50].

- United Kingdom

Improving the cancer patients’ experiences is a priority for the National Health Service in the United Kingdom. As part of this, the management of care by oncology clinical nurse specialists is central to the National Health Service strategy. According to an English population-based study using linked data from the National Cancer Registration and Analysis Service and patient experience questionnaires by the National Cancer Patient Experience Survey, of 100,885 colorectal, lung, breast and prostate cancer patients who were diagnosed between 2010 and 2014, 91.4% received a referral to a named clinical nurse specialist in one of the first stages of diagnosis or treatment [27]. The researchers suggest that providing patients with a name for their oncology clinical nurse specialist allows a relationship to grow more quickly immediately post-diagnosis, which especially helps mitigate patient frustration with the Sisyphean process of describing their concerns and needs to a large number of clinicians during a difficult time in their life and that of their family. Alongside providing direct care for patients, oncology clinical nurse specialists in the United Kingdom have played an important role in improving cancer treatment services and speeding up the diagnosis process [30,51]. An oncology clinical nurse specialist who helped establish an emergency oncology service at the Royal Liverpool and Broadgreen University Hospital NHS Trust has stated that, “The patient is at the heart of what I do, whether it be providing information, explanations, holistic support, reassurance, and bringing together all disciplines to make an informed and timely plan for the patient and treating team” [52]. According to the National Cancer Patient Experience Questionnaire, referral to an oncology clinical nurse specialist increased the sense of involvement in treatment decisions by an odds ratio of 2.69 for colorectal cancer, 2.41 for lung cancer, 2.68 for breast cancer, and 2.11 for prostate cancer (adjusted odds ratios for age, ethnicity, area, socio-economic deprivation, route to diagnosis, stage, and in lung and colorectal, sex). For all types of cancer, there was a distinct effect of referral to an oncology clinical nurse specialist in terms of feelings around care coordination, being treated with respect and dignity, and improved feelings in relation to National Health Service treatment.

While specialist clinical nursing in oncology is not new in the United Kingdom, the responsibilities of oncology clinical nurse specialists have gradually expanded with the encouragement of the National Health Service, with the stated aim of improving patient experience. However, at the same time, there is a high degree of heterogeneity and often confusion [2,24] among oncology clinical nurse specialists. It seems that the United Kingdom’s Department of Health’s Cancer Reform Strategy [10] was the clearest recommendation to increase autonomy and flexibility in relation to the National Health Service and to generate innovation to improve patient choice of different services under the management of a medical center, which is one of the key recommendations in the document. A survey of 103 medical centers found that most (76%) served as clinical leads for cancer patients or (11.7%) would like to establish clinics for cancer patients [2]. Most of the oncology clinical nurse specialists in the survey provided treatment that had previously been provided by doctors, in particular prescribing drugs and giving diagnoses. It seems that even in hospital teams there is a great appreciation for nursing skills and support for increasing their powers, thereby reducing the burden for various specialist doctors [26]. The most notable achievement of the Cancer Reform Strategy and the National Health Service’s decision to put full trust in nurses are clinics managed by oncology clinical nurse specialists, resulting in reducing waiting times for oncology services and making treatment accessible to people who do not live close to major cities. Alongside the progressive approach and targeted innovation, there is an acknowledgement that many areas of the work of the oncology clinical nurse specialist are insufficiently regulated and are too open to interpretation. Few would argue that the flexibility and initiative taken by the National Health Service was not the right step in terms of its strategy to improve cancer survival rates, but drastic changes in oncology clinical nurse specialist responsibilities alongside a lack of supervision make it harder to monitor and evaluate the treatment that patients receive [2].

- Japan

In 1987, a Japanese government report described for the first time the “need to cultivate specialist nurses with specialist nursing knowledge” [53]. In the wake of the report, Japan’s Association of University Programs in Nursing and a number of nurses’ organizations established a training and certification system that began operating in 1995, covering a variety of specialties including oncology [6]. By 2010, 193 oncology-certified nurse specialists were qualified and trained in medical settings, a number that was deemed insufficient, in line with the World Health Organization’s assessment that Japan would soon have a shortfall of around 270,000 nurses [15].

Responsibility for the professional capabilities of oncology-certified nurse specialists is shared by the Association of Nursing Programs in University and the Association of Oncology Certified Nurse Specialists. After completing a master’s degree in nursing with a defined area of expertise, a clinical nurse can apply to the Association of Oncology Certified Nurse Specialists for certification after gaining 5 years of experience in clinical work, of which 3 years should be in oncology nursing. The certification grants a license for 5 years, after which the Association of Oncology Certified Nurse Specialists requires nurses to apply for renewal before a committee. As part of the process, nurses must also submit a report on their work and advanced training undertaken.

A survey examining job satisfaction among 200 oncology-certified nurse specialists in Japan found that factors that increased job satisfaction included: positive assessment by senior staff (OR = 13.15), independence at work (OR = 11.30), involvement in cross-sectional activities and roles (OR = 7.06), ability to charge a fee for additional pain relief management (OR = 3.78), work in radiotherapy (OR = 2.91), and work with palliative care teams (OR = 2.64) [44]. Forty-nine percent of oncology-certified nurse specialists surveyed expressed satisfaction with their work, compared to 38% of nurses who are not oncology-certified nurse specialists.

The shortfall in healthcare professionals in Japan notwithstanding, the relative success of the establishment of the oncology-certified nurse specialist profession is noteworthy within the cultural context of the Western Pacific region, where, according to the World Health Organization, countries award a limited range of professional qualifications and degrees to nurses compared to their European and North American counterparts [15]. Looking ahead, Japan’s aim is to increase the number of oncology-certified nurse specialist training programs to 100 programs in universities across the country [6].

- Brazil

Calls to deepen oncology knowledge among nurses in Brazil began to emerge in 1990, led by nursing students in nursing colleges. These calls led to the creation of the oncology clinical nurse specialist and the pediatric oncology clinical nurse specialist roles, in line with the “North American model” [54]. The official foundation of the oncology specialty in nursing is set out in Resolution no. 293/2004 of the Federal Board of Nursing in 2004 [55]. At least in some workplaces, the competencies defined by the Association of Pediatric Hematology and Oncology Nurses are used for establishing this role. These include direct patient treatment, nursing consultation, systemic leadership, collaboration with nursing teams and service recipients, mentorship, research and active involvement in ethical decision-making [56]. Adaptation of the role was undertaken with inspiration from, and in cooperation with, oncology clinical nurse specialists in hospitals in Canada and the United States [54].

To qualify for the role, Brazilian clinical nurses must pass exams on chemotherapy and central venous catheterization, have at least two years of relevant clinical experience, and be involved in advanced studies [55]. An oncology clinical nurse specialist is required to graduate with at least 4608 h of practical internship and 1152 h of practical and theoretical training in oncology. The required curricula include epidemiological and bioethical issues in oncology, conceptual bases and diagnostic tools used in oncology, therapeutic models, oncology pathologies, oncological emergencies, palliative care and symptom control, and management in oncology [55]. Compared to other low- and middle-income countries, Brazil, aided by the Federal Nursing Council, stands out in its commitment to assimilating the oncology clinical nurse specialist position [14]. A unique example of this commitment is the fact that Brazilian oncology clinical nurse specialists trained for this post are able to fit central venous catheters [57]. Aside from the existing infrastructure, it has been suggested that oncology clinical nurse specialist training in Brazil is insufficiently broad or comprehensive. Of the 420 nursing schools in southeastern Brazil, the most developed region of the country, just 31 offer specialist courses in oncology, and it is estimated that between 2005 and 2013, only 150 nurses were trained as oncology clinical nurse specialists [55].

- Australia

Specialist nursing roles in oncology have existed in Australia and New Zealand for many years [41]. However, in 2005, the Australian government began funding the National Cancer Nursing Education Project, which laid the groundwork for dividing Australia’s specialist cancer nursing workforce into specializations. In the process, the role of “specialist cancer nurse” was defined to work within an oncology service or center specializing in a specific type or treatment stage of cancer, or as nurses involved in coordinating treatment for patients throughout all stages of a specific disease, such as breast cancer [58], or prostate cancer [41]. In terms of educational requirements, it was determined that these specialist professions would require a master’s degree without the need for any additional approvals, but the authority to prescribe medication (and other powers) would only be granted with additional approval. A qualitative study in Australia and New Zealand that examined the perceptions of 66 staff members relating to teamwork with oncology clinical nurse specialists in the treatment of gynecological tumors found that they were a key component of teams. Furthermore, although these teams comprised a long list of medical professionals and specialist nurses, the oncology clinical nurse specialist was an almost irreplaceable focal point of “contact, communication, and coordination” [43]. In addition to the sense that gynecological cancer teams without an oncology clinical nurse specialist were deficient, shortcomings and concerns were also raised by teams that did have an oncology clinical nurse specialist, including the development of dependence on the specialist nurse, carrying out “inter-functional” tasks that dedicated staff members were capable of performing no less well, encroaching on the responsibilities of other professionals in a way that harmed the work of the team, and ambiguity of the role of the oncology clinical nurse specialist working within a multidisciplinary team and taking on burdensome tasks tirelessly and sometimes needlessly [43].

Australia is a large country, and patients in the provinces and rural areas suffer from poor access to specialist medical care and are required to travel large distances for cancer tests and treatments. At least some of the areas of expertise in oncology nursing were founded with the aim of creating flexibility in service provision to meet local needs outside of urban centers [14,42,59]. Since 2010, the Australian government has been operating rural oncology clinics under the management of specialist nurses, with the aim of bridging gaps in access to oncology services between urban and rural areas. A study that evaluated the effectiveness of this service in the state of Victoria assessed a team comprising a nurse practitioner and additional medical and nursing staff in the city of Bendigo, which is in continuous contact with a clinic in a nearby village with a population of around 1000. The nurse practitioner and the rest of the team travel by car for about 90 min every two weeks to manage treatment in the village, which is actually carried out by a nurse living in the village [11]. The study emphasized the good relationships and high level of trust between all those involved in the therapeutic model. The nurse practitioner said of the service, “we love it because the patients are grateful, it’s rewarding, and they get treatment closer to home” [11], although it is not certain if the service is financially sustainable [14].

## 4. Discussion

The literature review shows that in the United Kingdom, oncology clinical nurse specialists play an important role in the National Health Service in the effective implementation of numerous initiatives to improve cancer diagnosis and treatment services [60]. The oncology clinical nurse specialist role is seen as essential in the United Kingdom, and the authority of oncology clinical nurse specialists to respond quickly to identify and treat emerging medical conditions is seen as preventing hospitalizations and the need for emergency service involvement [9,60].

An analysis of oncology clinical nurse specialist care workloads in patients with lung cancer in the United Kingdom found a reduction in preventable hospitalizations for non-acute problems from an average of 4 hospitalizations per month to 0.3 [29]. A qualitative study in the United Kingdom found that in lung cancer care teams, lung cancer nurse specialists contributed to faster and more accurate therapeutic prescriptions [39]. A quasi-experimental study from South Korea found that patients treated by oncology clinical nurse specialists rate their experiences of pain and exhaustion during treatment as lower, and satisfaction with treatment and quality of life as higher, but no differences were seen in anxiety or unplanned hospitalizations [32].

An integrative literature review regarding the implementation of the oncology clinical nurse specialist role in South Korea, Australia, the Netherlands, Ireland, and the United Kingdom, identified that, overall, the evaluation of the role was positive. Patient-related outcomes to which oncology clinical nurse specialists contributed can be divided into five main areas: psychological support, providing information to the patient, symptom management, treatment coordination, and patient satisfaction [30].

Patients suffering from a variety of cancers [25] emphasize the notion of being given enough emotional support in the presence of oncology clinical nurse specialists, and appreciated being viewed in the context of their whole lives in contrast to a collection of symptoms. In clinics with fewer patients per oncology clinical nurse specialist, treatment was evaluated as being more successful in terms of controlling chemotherapy side effects. A study from the United Kingdom showed that spouses of patients treated by oncology clinical nurse specialists saw their nurse as a trusted person to whom they could turn with concerns and doubts about their loved ones in situations where they preferred not to burden their family members [36].

The large degree of flexibility in the definition of the oncology clinical nurse specialist’s role and the autonomy granted to them have previously been attributed to the denigration of the role, which has been criticized for inappropriate and wasteful use of human resources in the workplace. At the same time, these qualities address a real need for flexible responses and sensitivity to a variety of changing contexts, which are seemingly not addressed by any other role in the healthcare system [30].

The oncology clinical nurse specialist role is almost universally commended for its unique benefits to patient outcomes. However, recurring themes in the literature include workplace confusion regarding the scope of the role’s responsibilities, misunderstandings regarding the role by other nurses, professionals, and managers, and ambiguity pertaining to role implementation that can lead to difficulties in assessing outcomes that are associated with the role [2,24]. Additionally, it is this versatility of the role that has, at times, been thought to contribute to its misinterpretation, to the nonoptimal use of oncology clinical nurse specialists’ expertise [23], or to the role being described as an “unaffordable luxury” [61]. Conversely, some have argued that contemporary modalities of implementation of the oncology clinical nurse specialist role do not meet patient needs in a sufficiently comprehensive way [62].

A mixed methodology study in Ireland indicated that 59% of women with breast cancer who were treated by oncology clinical nurse specialists felt that they did not receive sufficient information about their nutritional needs, a phenomenon later explained as stemming from a lack of personnel and ambiguities in the definition of the oncology clinical nurse specialist role [37]. The ambiguities regarding the areas of responsibility of oncology clinical nurse specialists within multidisciplinary teams have the potential to create tensions and challenges due to their overlap with a variety of professions [43]. It is therefore desirable to clearly define “the boundaries of the role” [30]. The planning, execution, and evaluation of studies is a necessary component in defining the role of the oncology clinical nurse specialist. However, there is doubt as to whether sufficient care is taken to include this factor when planning oncology clinical nurse specialist roles [30,63].

Given that the oncology clinical nurse specialist fulfills multiple, diverse, and multifaceted needs that are sometimes hard to define and quantify, evaluating this role is particularly challenging [4]. The systematic collection of health outcomes related to the work of the oncology clinical nurse specialist is important if we are to correctly assess the contributions of the role to the bigger picture and to ensure proper use of resources [35].

### 4.1. Improving Training

According to Kagan [38], today’s oncology clinical nurse specialists (and oncology nurses in general) are not adequately qualified to deal with an aging population suffering from cancer. In light of global demographic trends, clinical expertise in oncology must incorporate gerontological or geriatric components in future, since many elements of oncology nursing, including coordinating between various treatments and implementing behavior change, are becoming increasingly challenging as populations age [38].

In terms of pediatric oncology, it has been reported in Brazil that training is not precise enough, since there is no supervision of the formal education provided to oncology clinical nurse specialists. There is a countrywide shortfall of pediatric oncology courses, and the relevant knowledge within the nursing community caring for this population is incomplete [55]. Training provided to oncology clinical nurse specialists regarding cytotoxic substances is also insufficient, and may expose them to occupational risks [14]. Professional training in handling these substances exists in just 27% of Eastern European countries and 65% of Western European countries. Similarly, nurses from just 39% of European countries reported being given written instructions on handling radioactive materials.

To support oncology clinical nurse specialists starting out in the role, and to enhance their autonomy, there is value in network-based training programs similar to those in the United States. Distance training with digital aids has the potential to leverage the unique qualities of the oncology clinical nurse specialist [7]. In general, a structured, uniform training and implementation model for oncology clinical nurse specialists will empower nurses, benefit patients, and reduce the burden on oncology doctors, especially in peripheral areas. In-depth consideration is needed regarding the content of training for this role, as well as its boundaries and its assimilation. Such consideration should be made in full cooperation with oncologists and relevant professional organizations, to avoid creating unnecessary tensions and conflicts in the oncology profession.

### 4.2. Research and Practice Implications

It is recommended that further research be conducted to explore and quantify the specific gains and benefits of the oncology clinical nurse specialist role for cancer patients, oncology physicians, and the healthcare system. This could involve examining patient outcomes, cost-effectiveness, patient satisfaction, and quality of care. Additionally, investigating the challenges and barriers associated with the implementation of the role would provide valuable insights for effective integration into healthcare systems. Comparative research across different healthcare settings could also help identify the best practices for implementation.

To enhance the implementation and impact of the oncology clinical nurse specialist role, the establishing of official regulations or guidelines by relevant authorities, such as the Ministry of Health, is recommended. This would help define and recognize the role’s authority and boundaries, ensuring consistency and clarity in its implementation. Simultaneously, it is beneficial to foster interdisciplinary collaboration and dialogue between oncology clinical nurse specialists and other oncology professionals as a vehicle for the promotion of better teamwork and efficient working methods.

## 5. Conclusions

The oncology clinical nurse specialist role offers myriad gains for cancer patients, oncology physicians and the healthcare system. This is a necessary role, albeit one that brings specific challenges with its implementation. To maintain the flexibility and autonomy that is integral to this role, it is recommended that its recognition and the limits of its authority are set out in official regulations by the Ministry of Health. We believe it is futile for organizational leaders to shy away from potential tensions associated with the introduction of the oncology clinical nurse specialist role, and that respecting the ability of professionals to find creative solutions and negotiate workflows that are effective at a local level will maximize the mandate of the role while allowing continuous merit-based growth. Healthcare policymakers must intervene and facilitate dialogue between oncology clinical nurse specialists and other oncology professionals to engender creative cross-professional dialogue, without detracting from the ability of professionals to resolve tensions at a local level. It is important to recognize that it is in the best interests of both cancer patients and healthcare systems that caregivers cooperate as teams to create “new and efficient ways of working”, commensurate with their professional responsibilities.

## Figures and Tables

**Table 1:** PCC framework for identifying the main concepts of the rapid review.

PCC Element	Definition
Participants	National healthcare systems with established oncology clinical nurse specialist roles or oncology advanced practice nurse roles
Concept	Role implementation characteristics, including training and licensing/accreditation, background to and evaluation of role introduction
	Barriers and difficulties during or following role implementation
Context	Research publications after December 2001

**Table 2:** Non-observational and observational studies by type.

**Reviews**				**Observational Studies**			
Systematic Reviews		Other Reviews		Small and Experimental Design		National Studies	
Author	Year	Author	Year	Author	Year	Author	Year
Glover et al. [23]	2006	Raja-Jones [24]	2002	Bryant-Lukosius et al. [5]	2007	Griffiths et al. [25]	2013
Bryant-Lukosius et al. [12]	2010	Corner [9]	2003	Ream et al. [26]	2009	Alessy et al. [27]	2021
DiCenso et al. [19]	2010	Brooten et al. [28]	2004	Baxterand Leary [29]	2011		
Kerr et al. [30]	2021	Kagan [31]	2008	Farrell et al. [2]	2011		
		Komatsu [6]	2010	Kim [32]	2011		
		Kleinpell et al. [33]	2019	Rosenzweig et al. [34]	2012		
		Balsdon and Wilkinson [35]	2014	Borland et al. [36]	2014		
		Challinor et al. [17]	2016	Droog et al. [37]	2014		
		Kagan [38]	2016	Tod et al. [39]	2015		
		Morgan and Tarbi [40]	2016	Kilpatrick et al. [20]	2016		
		Challinor et al. [14]	2020	Wall and Rawson [18]	2016		
		Fleure and Sara [41]	2020	Crawford-Williams et al. [42]	2018		
		Young et al. [13]	2020	Galassi et al. [16]	2018		
				Hoffman et al. [7]	2018		
				Cook et al. [43]	2019		
				O’sullivan et al. [11]	2019		
				Kitajima et al. [44]	2020		
